# Early tolerance mechanisms in citrus: transcriptome and hormone profiling of NPR1-mediated responses to *Candidatus* Liberibacter asiaticus

**DOI:** 10.3389/fpls.2025.1719694

**Published:** 2026-01-15

**Authors:** Poulami Sarkar, Chunxia Wang, Amit Levy

**Affiliations:** 1 Citrus Research and Education Center, University of Florida, Lake Alfred, FL, United States; 2 Department of Entomology, University of California, Riverside, CA, United States; 3 Department of Plant Pathology, University of Florida, Gainesville, FL, United States

**Keywords:** Liberibacter, citrus, NPR1, transcriptome, immunity

## Abstract

Huanglongbing (HLB), caused by *Candidatus* Liberibacter asiaticus (CLas), is the most destructive citrus disease worldwide, severely reducing yield and fruit quality. Although no naturally resistant cultivars are available, citrus plants overexpressing *Arabidopsis* NPR1 (*At*NPR1) display enhanced tolerance, yet the molecular mechanisms underlying this phenotype remain insufficiently understood. To uncover early transcriptional events associated with tolerance, we performed comparative RNA-seq and phytohormone profiling of susceptible wild-type (WT) and tolerant *At*NPR1-overexpressing (*At*NPR1-OE) Duncan grapefruit at 0, 6, and 24 hours post infection (hpi). Before infection, *At*NPR1 plants downregulated genes involved in cytoskeleton organization, cell wall biogenesis, and receptor signaling, suggesting a primed basal defense state. After CLas exposure, *At*NPR1 plants exhibited stronger and earlier transcriptional reprogramming, with substantially more differentially expressed genes at 6 hpi than WT. At 24 hpi, *At*NPR1 plants showed suppression of callose synthase genes and selective induction of β-1,3-glucanases, indicating more controlled phloem callose regulation. Concurrently, attenuated expression of respiratory burst oxidase homologs and ROS-associated genes suggested a moderated and less damaging oxidative burst. *At*NPR1 plants maintained stable levels of salicylic acid, and gibberellins while preventing the CLas-induced induction of abscisic acid observed in WT. Collectively, these findings reveal that *At*NPR1 overexpression enhances HLB tolerance by integrating early transcriptional reprogramming with balanced structural, oxidative, and hormonal responses. This study provides a mechanistic framework for understanding NPR1-mediated tolerance to CLas during the initial stages of infection.

## Introduction

Citrus greening or Huanglongbing (HLB), associated with *Candidatus* Liberibacter asiaticus (CLas) is the most devastating citrus disease worldwide. HLB has reduced the production of citrus in Florida with diminished fruit quality up to 74%, leading to huge production costs (USDA). Citrus tissues infected with HLB exhibit a progressive degeneration of the phloem tissue with increased deposition of callose and phloem protein plugs. This results in the blockage of photo-assimilates movement from source leaves to sink tissue (Schneider, 1968), resulting in mottling of leaves, chlorosis, vein corking, and most importantly unmarketable lopsided fruit production with premature fruit drop (Schneider, 1967). HLB-infected plants have reduced growth, thinner canopies, twig dieback and high accumulation of starch and defense hormones (Schneider, 1968; Etxeberria et al., 2009; Achor et al., 2010; Fan et al., 2013; Neupane et al., 2023; Hendrich et al., 2025). A hallmark of this disease dysregulation is the alteration of phytohormone networks which causes reduced growth, stomatal closure, and reduced nutrient uptake. CLas also induces widespread transcriptional reprogramming in citrus plants. There are multiple transcriptomic reports comparing WT-citrus plants with infected WT plants which depict high regulation of sugar and starch metabolism with downregulation of photosynthesis (Martinelli et al., 2012; Chin et al., 2021; Lombardi et al., 2024). Susceptible varieties like Washington navel have lower micronutrient concentration with more micronutrients accumulated in tolerant varieties such as Lisbon lemon (Chin et al., 2021). Overall, CLas-infection disrupts the basal immune response in susceptible citrus plants by the excessive production of reactive oxygen species (ROS) and callose, and by disrupting the hormone gibberellin, which eventually induces phloem cell death and HLB damage. In the absence of HLB-resistant cultivars, it is necessary to develop sustainable methods to protect the HLB-threatened citrus industry. There are various strategies implemented to combat this disease, such as antibiotic treatments (Archer and Albrecht, 2023; Archer et al., 2023; Albrecht et al., 2025), using trap crops (Tomaseto et al., 2019; Hallman et al., 2024), application of hormones (Singh et al., 2022), and developing transgenic varieties overexpressing immune related genes (Zhang et al., 2010; Dutt et al., 2015; Robertson et al., 2018; Qiu et al., 2020; Soares et al., 2022).

NPR1 (NON-EXPRESSOR OF PATHOGENESIS RELATED 1) is a key component in immune related tolerance mechanisms and is involved in SA-mediated signaling for systemic responses. Activation of NPR1 is tightly controlled by the cellular redox state; following pathogen-induced accumulation of salicylic acid (SA), NPR1 undergoes conformational changes that allow its translocation to the nucleus. Once in the nucleus, NPR1 interacts with members of the TGA family of transcription factors to induce the expression of pathogenesis-related (PR) genes and other defense-associated transcripts. Through this regulatory network, NPR1 integrates SA signaling and coordinates broad-spectrum and long-lasting resistance responses against diverse pathogens (Zhou et al., 2023). Transgenic citrus plants over-expressing *Arabidopsis NPR1* (*AtNPR1*) gene have been reported to have enhanced tolerance to HLB (Robertson et al., 2018) and have a characteristic balanced immune response against CLas infection without allowing increased callose deposition and ROS generation. *AtNPR1*-overexpressing (*AtNPR1*-OE) citrus plants have inherently elevated basal levels of callose in the phloem cells, but suppresses over accumulation of callose and ROS following CLas infection. Transcriptomic reports comparing *AtNPR1*-OE and WT- citrus plants reveal that NPR1 induces PR gene, pathogen-associated molecular patterns, WRKY gene, and hormone-regulation pathway-gene expressions (Dutt et al., 2015; Qiu et al., 2020; Gao et al., 2023). Since NPR1 also mediates extensive crosstalk between salicylic acid and other hormones such as jasmonate, ethylene, auxin, and gibberellins, its role may extend beyond immune activation to broader hormone regulation during pathogen challenge (Zavaliev and Dong, 2024). Among the hormones implicated in HLB responses, abscisic acid (ABA) plays a central role by promoting stomatal closure and callose deposition, processes that restrict pathogen spread but also exacerbate phloem plugging and assimilate transport blockage (Adie et al., 2007; Lim et al., 2015). In contrast, salicylic acid (SA), jasmonic acid (JA), ethylene, and auxin are more directly associated with defense activation and signaling crosstalk. SA signaling shows contrasting responses in citrus and is often suppressed in tolerant varieties after CLas infection, despite higher basal levels compared to susceptible ones (Folimonova et al., 2009). In Persian lime, SA increases upon infection but remains lower than in susceptible species, accompanied by benzoic acid accumulation (Pérez-Zarate et al., 2025). In contrast, gibberellic acid (GA) is essential for maintaining growth, improving yield, and reducing fruit drop, and its exogenous application has been used to alleviate HLB symptoms in citrus (Ma et al., 2022; Shahzad et al., 2024). However, the contribution of hormone signaling to *At*NPR1-mediated HLB tolerance remains unexplored.

Several reports describe the early host response in WT citrus plants to CLas exposure mentioning early downregulation in photosynthesis related pathways and late burst in some important defense-related genes including ATP biosynthetic and glycolytic pathways (Wei et al., 2021; Alves et al., 2025). Based on these findings, we carried out comparative transcriptomic and selective phytohormone analysis with healthy and infected *AtNPR1*-OE transgenic plants and susceptible wild-type (WT) plants to determine the molecular factors behind the tolerance mechanism during early infection status. Here, in this study, we compared susceptible WT with tolerant *At*NPR1-OE plants at three different time points of 0 hpi, 6 hpi and 24 hpi. This study identifies several important differentially expressed genes related to plant stress, immunity and hormone regulation regulated by *At*NPR1 overexpression. Our findings highlight the importance of NPR1 in regulating the immune deficiency in susceptible wild-type cultivars.

## Materials and methods

### Plants and insects used

Two-months old Duncan grapefruit plants (grown in the greenhouse with controlled temperature of 25°C and 60% relative humidity) of each WT and *At*NPR1-OE were used for this experiment. CLas-free and CLas-infected psyllids were reared in growth chambers (with 16h light, 25°C and 60% relative humidity) in individual cages. The colonies were tested for the presence of CLas every month as described in (Sarkar et al., 2025).

### Experiment set-up

A single medium-aged leaf of each plant (still connected to the plant) was put in a plastic jar with dome lids and ten CLas-free or CLas-infected psyllids were released into each cage. This experiment was carried out in the laboratory conditions with 25°C temperature and 16h light. Leaf samples were harvested and snap frozen from the plants at 0 hpi (hour post infection, 6hpi and 24 hpi, followed by RNA extraction using Qiagen RNeasy kit (Qiagen, MD). Total of four replicates were used, for each WT-uninfected, WT-infected, *At*NPR1-uninfected, and *At*NPR1-infected.

### RNA sequencing and differential expression analysis

Samples were collected at three time-points: 0 hpi, 6 hpi, and 24 hpi, with four biological replicates for each plant group. Total RNA was used for library construction with the NEBNext Ultra II Directional RNA Library Prep Kit (New England Biolabs, catalog #E7760) following the manufacturer’s instructions. Sequencing was performed on the Illumina NovaSeq 6000 platform. Clean reads were mapped to the Citrus sinensis Huazhong genome assembly (v1.0; http://citrus.hzau.edu.cn/index.php) using the corresponding annotation files from the Citrus Genome Database (https://www.citrusgenomedb.org/citrus_downloads/Citrus_sinensis/C.sinensis_Hzau_v1.0_genome/annotation/). Gene IDs were functionally annotated using EggNOG-mapper v2 against the EggNOG v5.0 database. Differentially expressed genes (DEGs) were identified using the DESeq2 package in R, with pairwise comparisons conducted for each time point and between WT and *At*NPR1 plants. Analyses were performed at the University of Florida’s HiPerGator high-performance computing cluster and in RStudio.

### Gene Ontology annotation and enrichment analysis

DEGs were subjected to Gene Ontology (GO) enrichment analysis to identify overrepresented biological processes. GO enrichment was performed in RStudio (version 4.3.2) using the topGO package. Fisher’s exact test was used to identify significantly enriched GO categories, with a significance threshold of p ≤ 0.05. Each DEG set from the respective comparisons was analyzed separately, using the full annotated transcriptome as the background gene universe. Visualization of enrichment results was carried out in RStudio using the ggplot2 package to generate bubble plots, where bubble size reflected the number of DEGs per GO term and color indicated statistical significance (–log_10_ p-value).

### Comparative analysis

Comparisons between time points for infected samples and between WT and *At*NPR1 plants and were conducted using TBtoolsII (Chen et al., 2020) and RStudio.

### Hormone detection using liquid chromatography-mass spectrometry

80–100 mg of leaf samples were harvested from each plant at 24hpi, snap-frozen in liquid nitrogen and homogenized using the TissueLyserII (Qiagen). Phytohormones were extracted from the tissue samples using cold methanol:acetonitrile (50:50, v/v) spiked with deuterium-labeled internal standards (mixture of D4-SA, ABA, JA-Ile and GA8). The samples were centrifuged at 16,000 g, and the pellets were allowed to dry and were resuspended in 15% methanol and run using a LC-MS MRM (Multiple Reaction Monitoring) targeted assay, as previously mentioned in (Lopez-Guerrero et al., 2022). The LC separation was done on a ZORBAX Eclipse Plus C18 column (2.1 mm × 100 mm, Agilent) flowing at 0.45 mL/min. All four phytohormones were detected using MRM transitions that were optimized using the standard. For quantification and analysis, an external standard curve was prepared using a series of standard samples having different concentrations of unlabeled hormones and fixed concentrations of the deuterium-labeled standards mixture.

## Results

### Morphological differences in the samples

**Figure 1:**
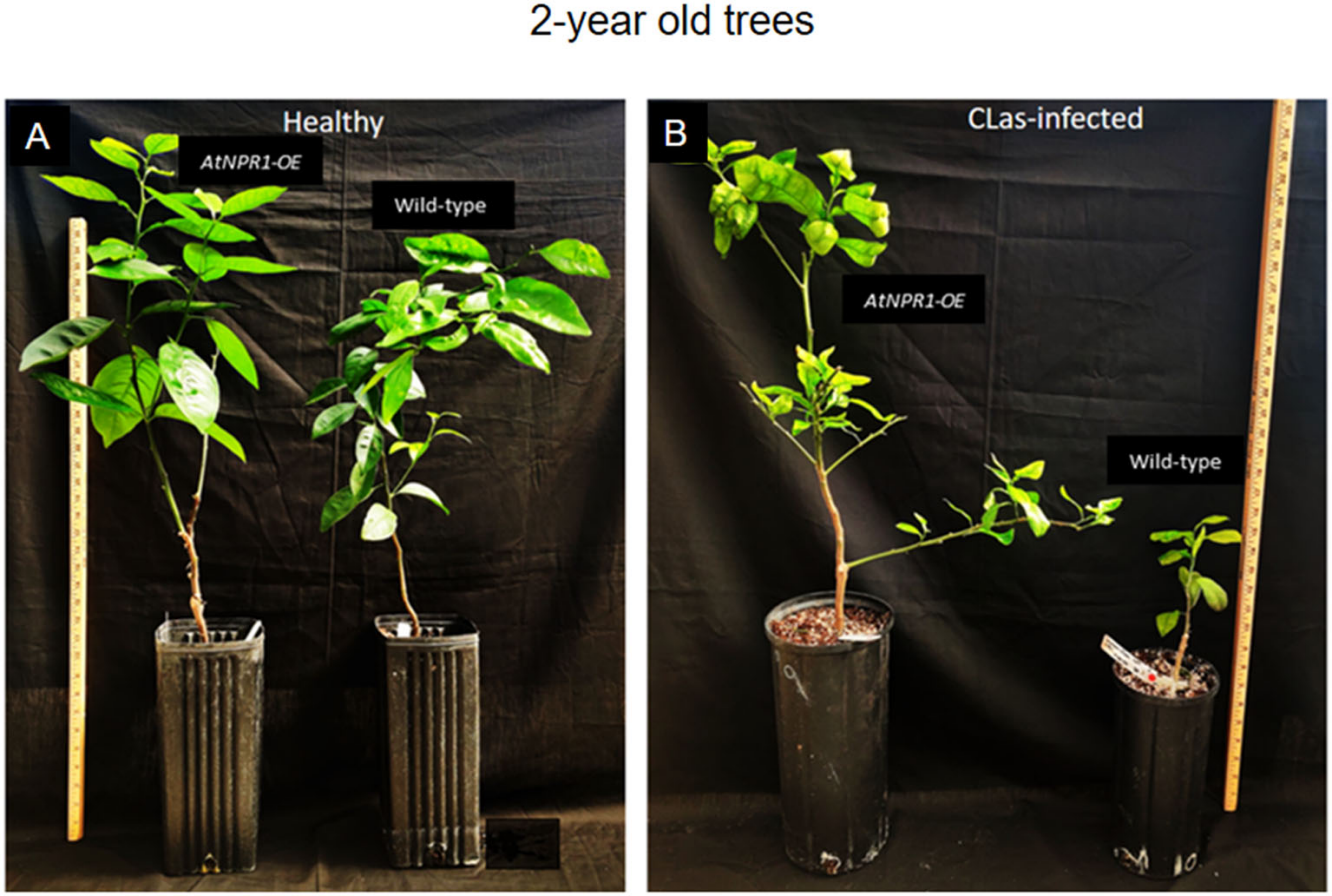
Greenhouse-grown WT and *At*NPR1 plants, healthy **(A)** and CLas-infected **(B)**. Two-year-old plants showing the impact of CLas infection. Infected WT plants exhibit reduced growth and pronounced leaf damage, whereas *At*NPR1 plants display comparatively healthier growth and less visible leaf symptoms.

Both susceptible WT and tolerant-transgenic *At*NPR1 plants were inoculated with CLas-infected psyllids for a month, and the plants were tested for CLas. Once infected, the plants were maintained in the greenhouse. CLas-infected (2-year-old) transgenic *At*NPR1 Duncan grapefruit plants displayed only mild symptoms of HLB compared to the severe decline observed in infected wild-type (WT) plants grown under greenhouse conditions. Following infection, WT plants were markedly shorter, exhibited poor growth, and developed pronounced leaf curling and chlorosis. In contrast, *At*NPR1 plants maintained normal growth and overall plant vigor, showing little to no visible deterioration upon CLas infection (**Figure 1**).

### RNA-seq and sample clustering

Leaves of WT and *At*NPR1 transgenic plants were harvested at different time points, and the midribs were used for RNA sequencing using Illumina platform (four replicates each). Sequencing yielded between ~47 and ~65 million raw paired end reads per sample. After quality filtering, nearly all reads were retained as clean reads. Mapping was performed against *Citrus sinensis* reference genomes: the Huazhong genome assembly (http://citrus.hzau.edu.cn/index.php). Uniquely mapped reads ranged from 36.7 to 56.6 million per sample, with unique mapping rates ranging from 77.3% to 87.5%. Multiple mapping rates were low (1.7%–8.6%), and unmapped reads accounted for less than 10%. Splice junction counts ranged between ~23M and ~50M, reflecting robust transcriptome coverage.

### Screening differentially expressed genes and annotation

**Figure 2:**
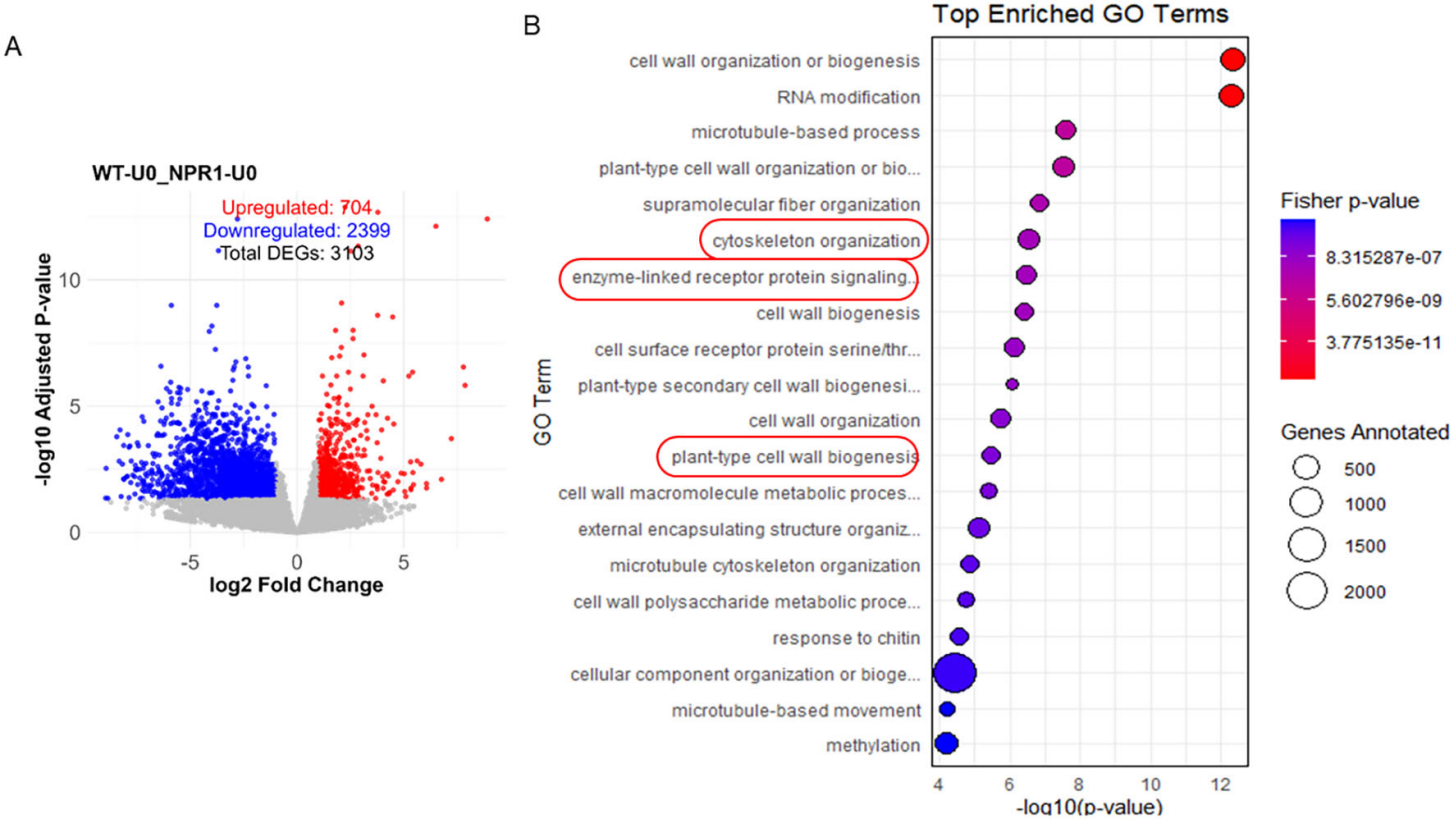
**(A)** Volcano plot of DEGs between A*t*NPR1 and WT plants at 0 hpi. The x-axis represents the log₂ fold change (log_2_FC) in gene expression, and the y-axis represents the –log_10_ adjusted p-value. Red dots indicate significantly upregulated genes in AtNPR1 relative to WT, blue dots indicate significantly downregulated genes, and grey dots represent genes with no significant differential expression. A total of 704 genes were upregulated and 2,399 genes were downregulated in AtNPR1 plants compared to WT. **(A)** Top enriched GO terms for DEGs between AtNPR1 and WT plants at 0 hpi. Bubble plot showing the top enriched Gene Ontology (GO) biological process terms. The x-axis represents the –log_10_ of the Fisher’s exact test p-value, with color indicating significance (red = more significant, blue = less significant). Bubble size corresponds to the number of genes annotated to each GO term. Enriched categories include cytoskeleton organization, cell wall biogenesis, and enzyme-linked receptor protein signaling, with most corresponding genes downregulated in AtNPR1 plants relative to WT.

Differentially expressed genes between healthy and infected samples were identified by DESeq2-based R pipeline, followed by its annotation by GO (Gene ontology) analysis. At time 0, more DEGs were downregulated in *At*NPR1 when compared to WT (2399) (**Figure 2A**). GO enrichment analysis of DEGs between *At*NPR1 and WT plants at 0 hpi confirmed that 77% of DEGs in the *At*NPR1 transgenic plants were downregulated compared to WT, including those associated with cytoskeleton organization, cell wall biogenesis, and enzyme-linked receptor protein signaling (**Figure 2B**).

**Figure 3:**
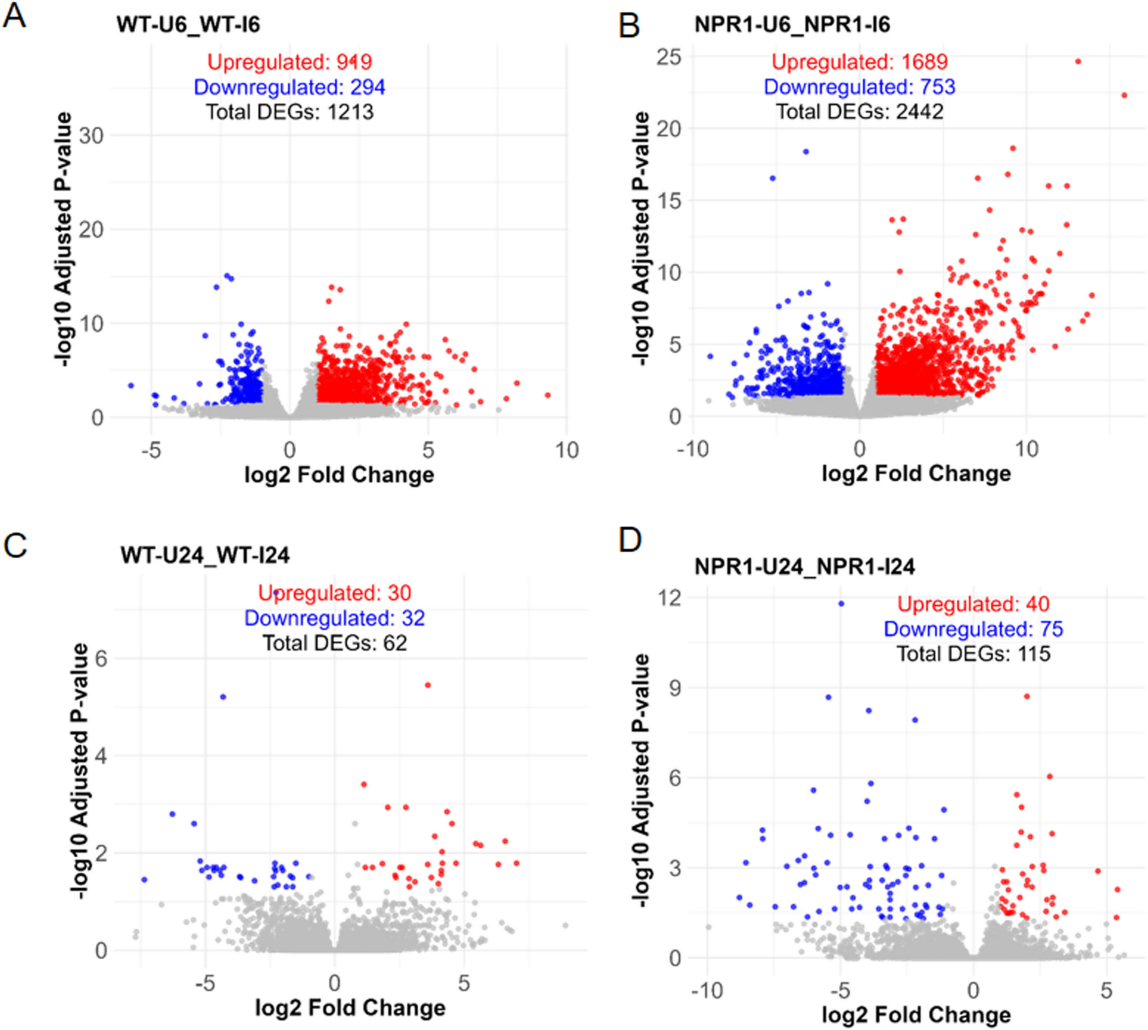
Volcano plots of DEGs between different time points in WT and AtNPR1 plants following CLas inoculation. **(A)** WT at 6 hpi, **(B)***At*NPR1 at 6 hpi, **(C)** WT at 24 hpi, and **(D)***At*NPR1 at 24 hpi. Blue dots represent downregulated genes; red dots represent upregulated genes. Significant DEGs were defined as those with an adjusted p-value (padj) ≤ 0.05 and log_2_ fold change (log_2_FC) > 1 or < –1. Overall, *At*NPR1 plants exhibited a greater number of DEGs at both 6 hpi and 24 hpi compared to WT, with a predominance of upregulation at 6 hpi and downregulation at 24 hpi, indicating a rapid and transient transcriptional response to CLas infection.

In *At*NPR1 plants, the number of DEGs was higher at both 6 hpi and 24 hpi compared to WT plants, although fewer DEGs were detected at 24 hpi relative to 6 hpi (**Figure 3**). Notably, the majority of DEGs at 6 hpi were upregulated in both *At*NPR1 and WT plants, whereas at 24 hpi, most DEGs were downregulated (**Figure 3**).

### GO enrichment for WT and *At*NPR1 plants

**Figure 4:**
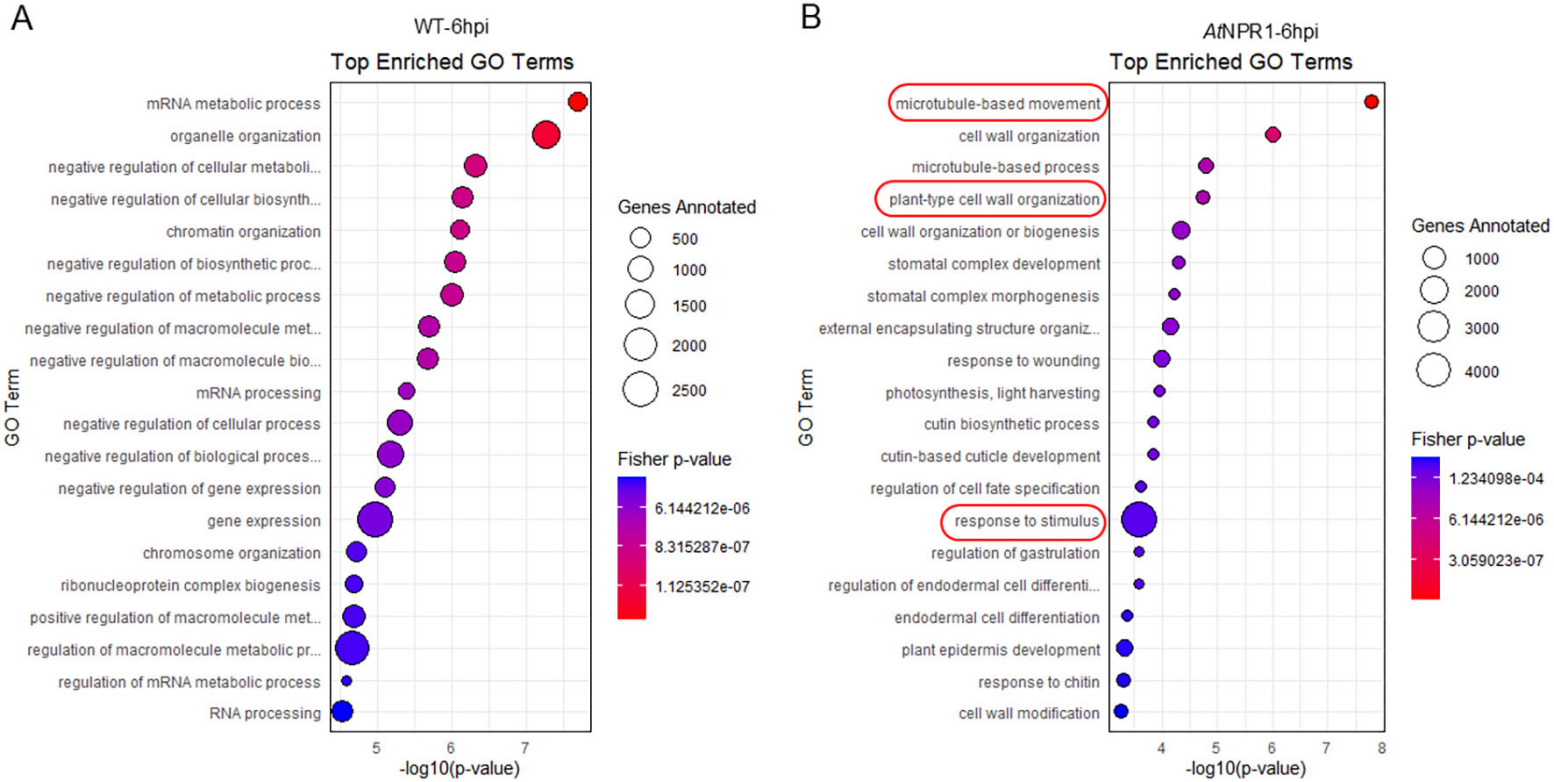
GO enrichment analysis of differentially expressed genes at 6 hpi. Bubble plots show the top enriched GO terms in WT **(A)** and *At*NPR1 **(B)**. The x-axis indicates significance level (−log10 p-value), bubble size corresponds to the number of genes annotated, and color represents enrichment significance (Fisher’s exact test p-value). WT plants were enriched in RNA metabolism and regulatory pathways, while, *At*NPR1 plants were enriched in defense- and cell wall–related processes.

GO enrichment analysis revealed distinct biological processes enriched in *At*NPR1 vs. WT plants at both 6 and 24 hpi. WT-6hpi showed strong enrichment of mRNA metabolic process, RNA processing, and chromatin organization, along with several categories related to negative regulation of metabolic and biosynthetic processes. This suggests that, rather than engaging robust structural and defense responses, WT plants at 6 hpi predominantly activated transcriptional and post-transcriptional regulatory pathways (**Figure 4A**). By contrast, in *At*NPR1-6hpi, the most significantly enriched terms included microtubule-based movement, cell wall organization, and stomatal complex development, alongside defense-related categories such as response to wounding. These enrichments highlight early activation of cell wall remodeling, cytoskeletal dynamics, and structural defense responses in *At*NPR1 plants (**Figure 4B**).

**Figure 5:**
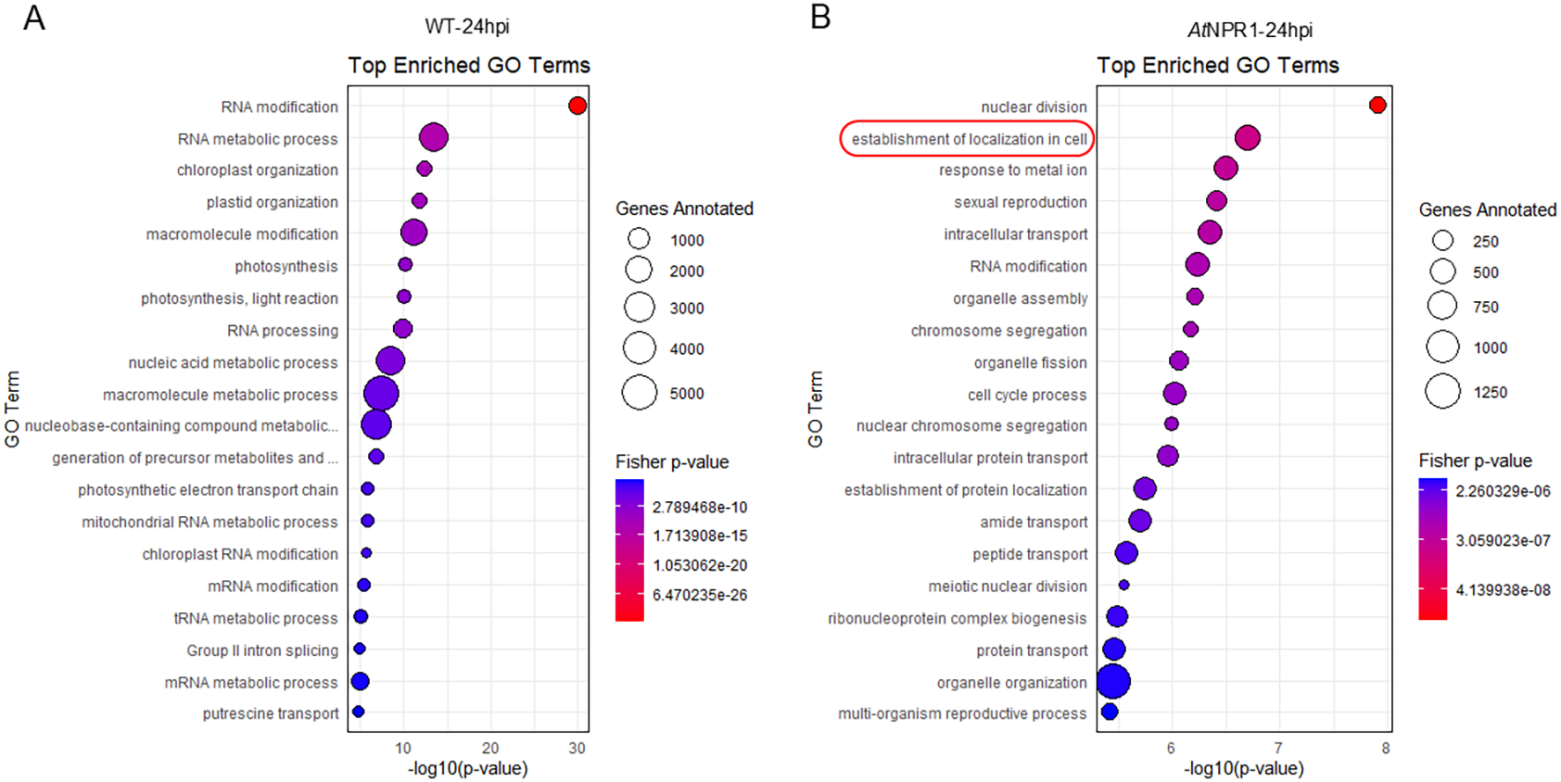
GO enrichment analysis of differentially expressed genes at 24 hpi. Bubble plots show the top enriched GO terms in WT **(A)** and *At*NPR1 **(B)**. The x-axis indicates significance level (−log10 p-value), bubble size corresponds to the number of genes annotated, and color represents enrichment significance (Fisher’s exact test p-value).

At 24 hpi, WT plants showed significant enrichment in RNA modification, chloroplast organization, and photosynthesis-related processes, suggesting a diversion of resources toward basal metabolic and energy-associated processes rather than immune activation (**Figure 5A**). Meanwhile, *At*NPR1 plants displayed enrichment of GO terms linked to nuclear division, cell cycle progression, intracellular transport, and protein localization, suggesting that *At*NPR1 modifies host transcriptional regulation to maintain cellular integrity under pathogen pressure (**Figure 5B**).

### DEG responses in WT and AtNPR1

**Figure 6:**
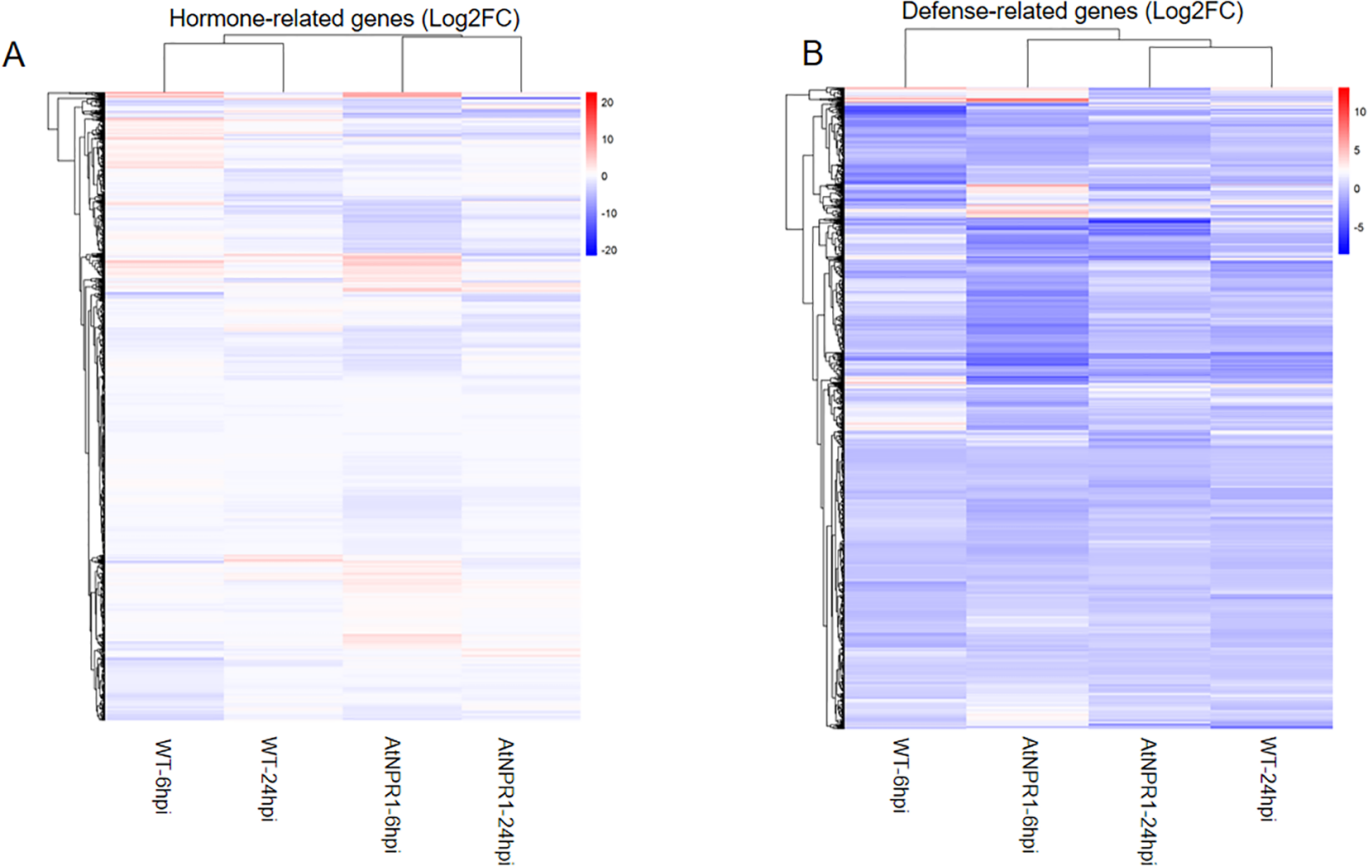
Heatmaps of category-specific differentially expressed genes. Heatmaps show log2 fold change values for hormone-related **(A)** and immune/defense-related **(B)**, genes across transgenic AtNPR1 and wild type (WT) plants at 6 hpi and 24 hpi. Colors represent relative expression changes (red = upregulation; blue = downregulation). Hierarchical clustering separates conditions, highlighting earlier and stronger activation of hormone and defense pathways in AtNPR1 plants, contrasted with stress/senescence enrichment in WT.

**Figure 7:**
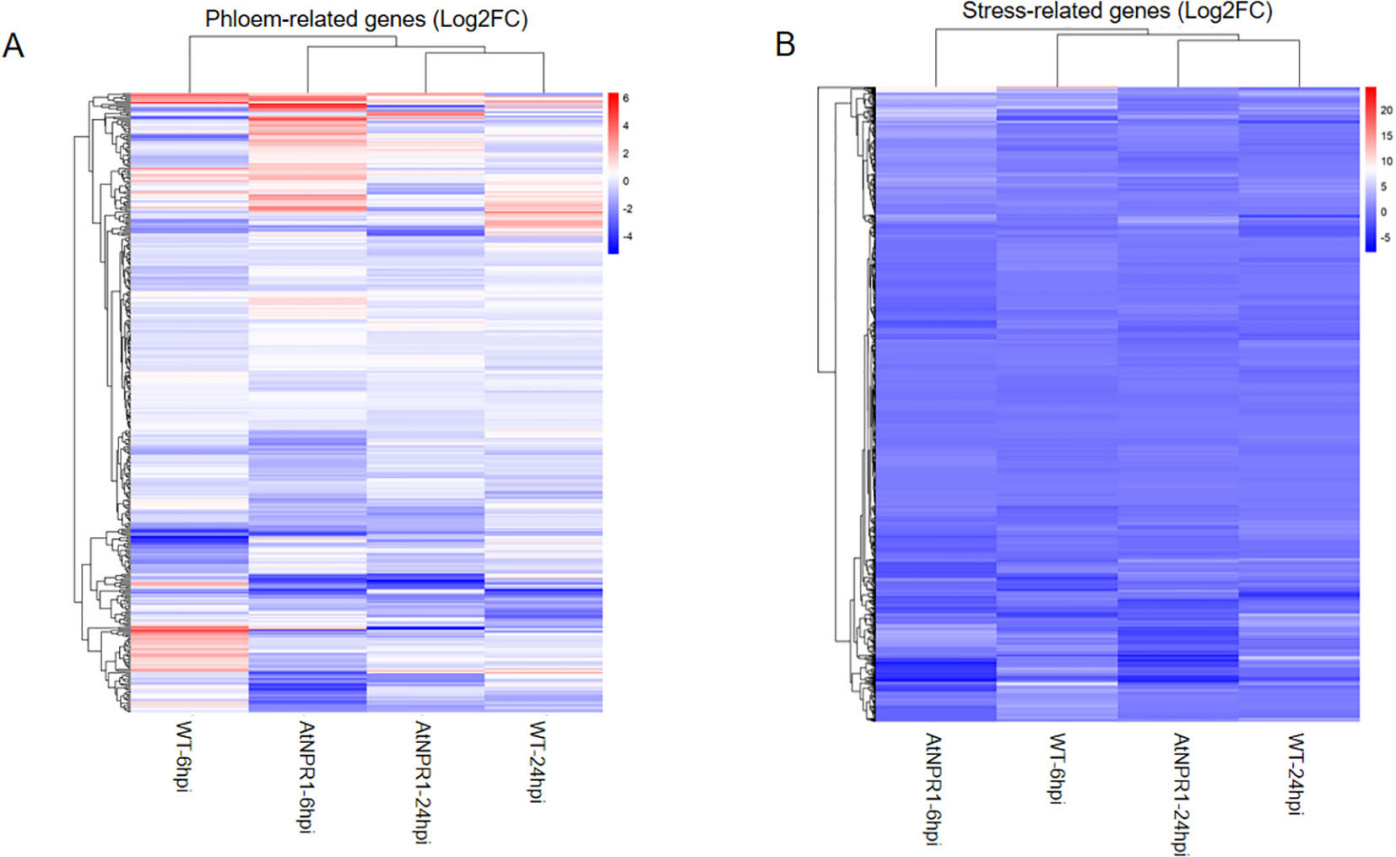
Heatmaps of category-specific differentially expressed genes. Heatmaps show log2 fold change values for phloem–sugar–callose-associated **(A)**, and stress/senescence-related **(B)** genes across transgenic A*t*NPR1 and wild type (WT) plants at 6 hpi and 24 hpi. Colors represent relative expression changes (red = upregulation; blue = downregulation). Hierarchical clustering separates conditions, highlighting earlier and stronger activation of hormone and defense pathways in A*t*NPR1 plants, contrasted with stress/senescence enrichment in WT.

To further investigate category-specific transcriptional responses, we generated clustered heatmaps of differentially expressed genes (DEGs) associated with hormones, immune/defense, phloem-related, and stress-related genes (**Supplementary Table 1**). The heatmap patterns highlight a tolerant transcriptional program in *At*NPR1 versus WT, but not much in stress-related genes (**Figures 6**, **7**).

#### a. DEGs involved in hormone regulation

Hormone-related genes displayed dynamic regulation across infection stages. Differential expression analysis of hormone-associated transcripts revealed clear differences between WT and *At*NPR1-overexpressing grapefruit following CLas infection. At 6 hpi, *At*NPR1 plants displayed strong upregulation of *ethylene-responsive transcription factors (ERF1, ERF109)*, *auxin-responsive proteins (IAA2, IAA9)*, and *gibberellin-regulated proteins*. In contrast, WT plants exhibited weaker induction of these regulators and instead showed repression of *auxin efflux carrier-like proteins (PIN1, PIN3)* and *ABA-responsive elements (ABI5)*. By 24 hpi, WT plants exhibited widespread repression of hormone signaling genes, including *jasmonate ZIM-domain proteins (JAZ1, JAZ6)*, whereas *At*NPR1 plants maintained elevated expression of *ERFs* and *auxin response factors (ARF5, ARF8)*.

#### b. DEGs associated with host defense

Several pathogenesis-related proteins (PR1, PR2, PR5) and *thaumatin-like proteins* were strongly induced in *At*NPR1 plants as early as 6 hpi but were suppressed in WT at both time points of 6 hpi and 24 hpi. Similarly, *lipid transfer proteins (LTP2, LTP5)*, *peroxidases (PRX34, PRX52)*, and *chitinase family members (CHIB, CHI4C)* were highly expressed in *At*NPR1, whereas WT exhibited sharp downregulation at 24 hpi. Key signaling components, such as *WRKY70* and *WRKY33*, showed *At*NPR1-specific induction, reinforcing the activation of SA-dependent defense pathways. The WT plants, however, showed reduced or delayed induction of these regulators, correlating with pathogen-driven suppression. Collectively, these results indicate that *At*NPR1 sustains a broad immune transcriptional program, spanning PR proteins, ROS-scavenging enzymes, and WRKY regulators, thereby countering pathogen-mediated immune suppression observed in WT. Beyond defense signaling, *At*NPR1 plants also displayed tighter control over phloem callose dynamics. Rather than initiating the excessive callose buildup commonly associated with HLB susceptibility, *At*NPR1 plants induced expression of glucanase genes (Cs2g20640) that degrade callose especially at 24hpi, helping maintain phloem transport during early infection. In WT, this regulatory balance was absent, aligning with their tendency toward uncontrolled callose occlusion.

#### c. Sugar transporter genes

Genes linked to carbohydrate transport were markedly altered. At 6 hpi, *At*NPR1 plants exhibited higher induction of *sucrose synthases (SUS1, SUS3)*, *sugar transporters (SWEET11, SWEET14)*. In contrast, WT plants repressed multiple *SWEET* transporters and *cell wall-associated kinases (WAK1, WAK2)*. At 24 hpi, WT plants showed further suppression of phloem stability genes, while *At*NPR1 maintained moderate induction of and *UDP-glucose transferases*, indicating sustained phloem function.

#### d. Senescence and stress response

Within the stress gene set, a large cluster of genes was directly associated with ROS signaling, redox regulation, and oxidative damage repair. Notably, thioredoxin-like proteins (e.g., Cs2g15600) and glutaredoxin family members were strongly induced in *At*NPR1 plants, supporting enhanced antioxidant buffering. Genes linked to DNA damage repair under oxidative stress, such as DNA-damage-repair/toleration proteins (Cs7g01980), were also enriched. In WT plants, several redox-associated transcripts, including oxidoreductases, catalase-like, and peroxidase family proteins, were suppressed at 24 hpi, reflecting a collapse of antioxidant defenses. Additional redox sensors such as receptor-like kinases (orange1.1t04678, orange1.1t05183, orange1.1t05299) were differentially regulated, further emphasizing genotype-specific ROS signaling dynamics.

### NPR1 overexpression alters hormone accumulation in citrus during early CLas infection

**Figure 8:**
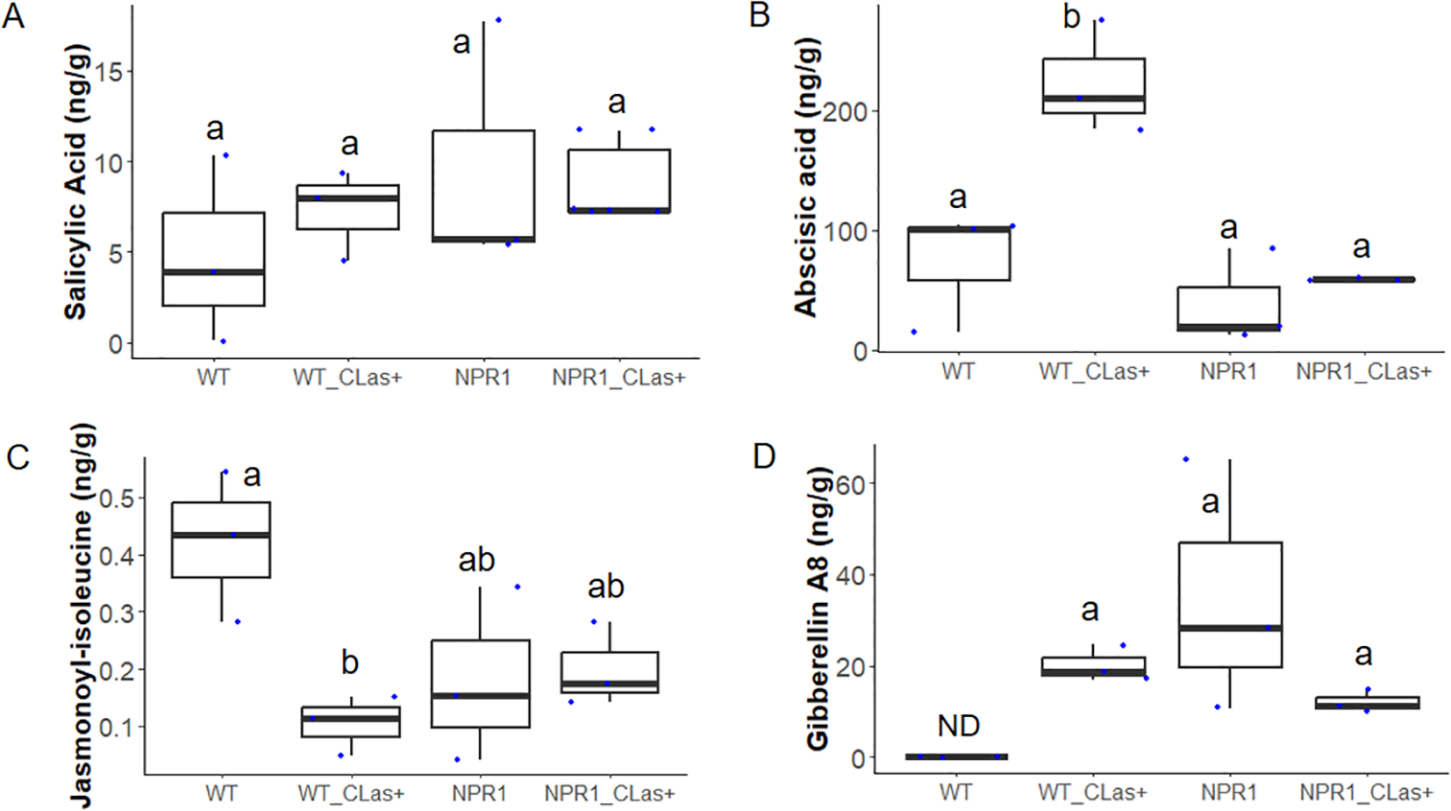
Hormone accumulation in WT and *At*NPR1-overexpressing citrus plants in response to CLas infection at 24 hpi. Levels of important hormones are depicted **(A)** Salicylic acid **(B)** Abscisic acid, **(C)** Jasmonoyl- isoleucine (JA-Ile), and **(D)** Gibberellin A8. Different letters represent statistical differences (p<0.05) with one-way ANOVA and *post-hoc* test. Each dot represents an individual biological replicate. Center lines of boxplots indicate medians. Statistical comparisons were performed using one-way ANOVA followed by Tukey’s *post hoc* test, with significance defined at *p* < 0.05. ND indicates not detected.

At 24 hpi, hormone profiling revealed distinct patterns of accumulation in wild-type (WT) and *At*NPR1-overexpressing citrus plants in response to CLas infection (**Figure 8**). Salicylic acid (SA) levels were comparatively elevated in *At*NPR1-overexpressing plants compared to WT (**Figure 8A**) and had no significant difference among the plant types following CLas infection. Abscisic acid (ABA) concentrations were significantly increased upon CLas infection in WT plants after CLas infection (**Figure 8B**), with no significant difference observed between *At*NPR1 plants. WT plants accumulated significant ABA within 24hpi. In contrast, jasmonoyl-isoleucine (JA-Ile) levels decreased in CLas-infected WT plants relative to uninfected controls, whereas *At*NPR1-overexpressing plants maintained stable JA-Ile levels regardless of infection status (**Figure 8C**) which was also noted in the RNAseq analyses (**Supplementary Table S1**). High JA-Ile signaling tends to suppress JMT expression, prioritizing local signaling (defense gene activation) over volatile emission (Li et al., 2022). Finally, gibberellin A8 (GA8) accumulation was not detected in susceptible WT plants with no infection, however *At*NPR1 plants have significantly higher amounts of GA8 without infection which tends to stay low following CLas infection (**Figure 8D**). WT plants upon infection revealed generous amounts of GA8 within 24 hpi consistent with the RNAseq results.

## Discussion

Overexpression of *Arabidopsis* NPR1 (*At*NPR1) has been shown to enhance tolerance in citrus (Zhang et al., 2010), yet the molecular basis of this effect has remained largely unexplored. In this study, we employed early transcriptome profiling coupled with phytohormone analysis to dissect the initial responses of susceptible wild-type (WT) grapefruit and *At*NPR1-overexpressing (*At*NPR1) lines to CLas infection. Prior to infection, *At*NPR1 plants displayed repression of genes (**Figure 2A**) associated with cytoskeleton organization, receptor signaling, and cell wall biogenesis, suggesting a primed defense state (**Figure 2B**). Such transcriptional changes may reflect reallocation of resources while maintaining readiness for rapid activation of defenses (Conrath et al., 2015). Upon infection, *At*NPR1 plants showed a stronger and earlier transcriptional response, with more differentially expressed genes (DEGs) at 6 hpi compared to WT (**Figure 3**). This rapid activation likely is linked to the immense immune response in the *At*NPR1 plants. *At*NPR1 plants may help preserve phloem function and maintain sugar transport, whereas WT plants displayed transcriptional trends consistent with pathogen exploitation of sugar flux through SWEET transporters (Breia et al., 2021). Reactive oxygen species (ROS) dynamics were also differentiated between the two genotypes. *At*NPR1 plants attenuated expression of respiratory burst oxidase homologs and ROS-responsive genes, correlating with a reduced oxidative burst (**Supplementary Table S1**). In WT plants, uncontrolled ROS accumulation is associated with oxidative stress, cell death, and symptom progression (Mittler, 2017). By moderating ROS production, *At*NPR1 likely prevents premature senescence and oxidative collapse, while still allowing ROS to function as defense signals. This aligns with prior evidence that NPR1 regulates cellular redox balance during immune responses (Zavaliev and Dong, 2024).

Callose regulation is another critical component of the *At*NPR1-mediated response to CLas. In citrus, phloem plugging via callose deposition is a hallmark of HLB pathology and is often associated with impaired photoassimilate transport and starch accumulation. Several phloem-associated genes linked to callose turnover displayed altered expression in our dataset. Callose synthases (Cs1g05830, Cs7g01200, Cs7g17355), remain tightly regulated in the *At*NPR1 plants (**Supplementary Table S1**). Notably, the endo-1,3-β-glucanase gene (Cs7g29260), a key enzyme responsible for callose degradation was reduced significantly in infected WT tissues but remained comparatively upregulated in *At*NPR1 lines. This pattern suggests that NPR1 may help maintain controlled callose turnover, preventing excessive sieve plate occlusion during early infection. Similarly, the xyloglucan-specific endoglucanase inhibitor protein (orange1.1t05612) was strongly induced in infected *At*NPR1 plants at 24 hpi, which could reflect a compensatory mechanism to fine-tune glucanase activity and avoid over-degradation of phloem structural polysaccharides.

Hormone profiling further highlighted NPR1’s role in coordinating defense. *At*NPR1 plants maintains elevated basal levels of salicylic acid (SA), a key activator of systemic acquired resistance (Durrant and Dong, 2004), while stabilizing jasmonoyl-isoleucine (JA-Ile) levels following infection. There was no significant difference in SA levels at 24 hpi (**Figure 8**), similar to *At*NPR1 plants at 14 days post-infestation (dpi) in previous studies where they exhibited slightly higher, but not significant, SA levels (Sarkar et al., 2025). This indicates that *At*NPR1 suppresses SA overaccumulation after CLas infection compared with WT plants. In WT plants, BSMT, a salicylic acid methyltransferase that converts SA into its inactive volatile form, methyl salicylate, was strongly induced (log_2_FC = 5) at 24 hpi (**Supplementary Table 1**). This delayed SA buildup likely influences the timing of oxidative and hormonal responses. In *At*NPR1 plants, reduced early expression of RBOH and related oxidative stress genes suggests a restrained or delayed oxidative burst, which may protect against premature tissue damage (Spoel et al., 2003; Maier et al., 2011). In contrast, *At*NPR1 plants showed no such induction in BSMT, suggesting that in WT, SA is rapidly methylated and detoxified early in infection, preventing its buildup. Reduced BSMT expression in *At*NPR1 plants likely allows SA to persist and accumulate later (14 dpi), explaining the delayed increase observed. On the other hand, WT plants exhibited infection-induced suppression of JA-Ile, consistent with CLas-mediated disruption of jasmonate signaling (Zhang et al., 2017). Pathogens are known to manipulate JA-Ile and exploit host defenses to establish infection (Zhang et al., 2017). Abscisic acid (ABA) concentrations increased significantly in WT plants but not in *At*NPR1 lines; as ABA is known to promote callose deposition (Kumar and Dasgupta, 2024), this may contribute to excessive plugging in WT phloem. Finally, gibberellin A8 (GA8) levels were maintained in *At*NPR1 plants, whereas WT plants accumulated GA8 only after infection (**Figure 8**), consistent with CLas manipulation of GA metabolism (Tadayon et al., 2023).

Collectively, these results indicate that *At*NPR1 fine-tunes hormone crosstalk, buffering SA-JA-GA interactions while preventing pathogen-driven exploitation of ABA and JA pathways. By maintaining SA at functional but non-toxic levels, stabilizing JA-Ile, and preventing excessive ABA and GA accumulation, *At*NPR1 ensures a coordinated defense signaling network that minimizes trade-offs between immunity and growth. This balanced regulation likely prevents the antagonistic suppression typically observed between SA and JA pathways in WT plants, allowing *At*NPR1 lines to sustain both localized and systemic resistance responses without triggering stress-induced senescence. Our findings further show that *At*NPR1 modulates phloem-associated defenses by influencing callose turnover, a central component of CLas-induced phloem plugging. In WT plants, elevated ABA and suppressed JA/GA signaling coincided with down-regulation of key β-1,3-glucanases and phloem-associated metabolic genes, consistent with excessive callose accumulation and impaired transport. In contrast, *At*NPR1 plants displayed a more controlled expression of callose-related enzymes, including maintenance of endo-1,3-β-glucanase transcripts and induction of a xyloglucan endoglucanase inhibitor, suggesting that NPR1 helps balance callose synthesis and degradation.

## Conclusion

**Figure 9:**
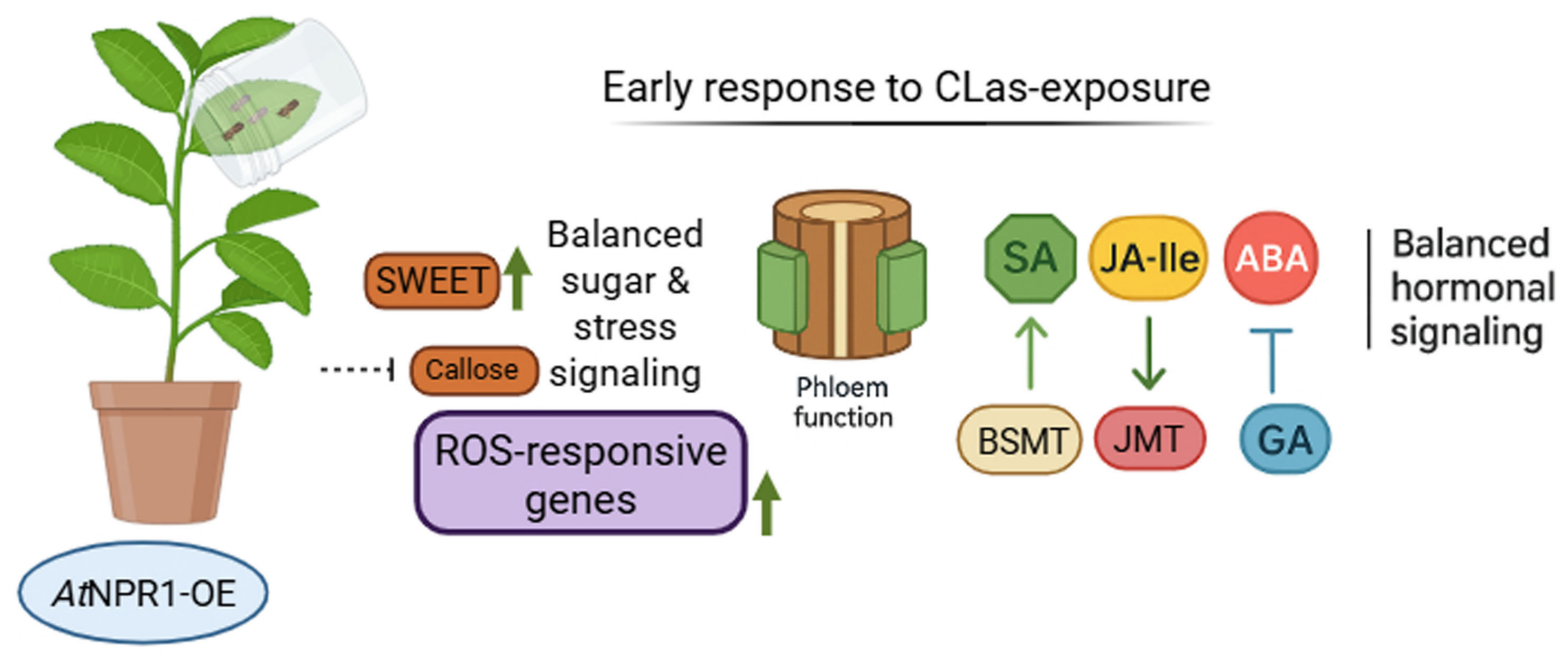
Early defense response of *At*NPR1-overexpressing (*At*NPR1-OE) citrus plants exposed to CLas-infected psyllids (on a leaf, in a dome cup). *At*NPR1-OEdisplay a coordinated transcriptional and hormonal response that promotes tolerance. NPR1 stabilizes salicylic acid (SA) with enhanced expression of BSMT (Benzoic Acid/Salicylic Acid Methyltransferase) which catalyzes methylation of SA to form methyl salicylate (MeSA) volatile long-distance signaling molecule, whereas jasmonoyl-isoleucine (JA-Ile) regulates JMT (Jasmonate o-methyltransferase) transcriptionally, as part of a feedback and amplification mechanism that enhances systemic and volatile signaling. NPR1 regulates gibberellin (GA) signaling for growth while preventing abscisic acid (ABA)-driven stress dominance. This balanced hormonal network enhances expression of BSMT and JMT, sustains sugar transport (SWEET), and regulates callose/glucanase dynamics in the phloem. In parallel, induction of stress-responsive and redox-buffering genes helps maintain cellular homeostasis. Together, these changes contribute to preserved phloem integrity, reduced pathogen-induced stress, and a tolerant phenotype against CLas. (Created with Biorender).

This study demonstrates that overexpression of *At*NPR1 in citrus orchestrates early and coordinated transcriptional, hormonal, and structural responses that collectively underpin enhanced tolerance to *Candidatus* Liberibacter asiaticus (CLas). In WT plants, CLas destabilized hormone homeostasis, suppressed JA- and GA-dependent pathways, elevated ABA levels, and promoted phloem obstruction through dysregulated callose deposition. By contrast, *At*NPR1-overexpressing plants rapidly activated defense pathways, maintained JA-Ile and GA metabolism, buffered SA-JA interactions, and stabilized redox signaling while preventing runaway callose accumulation. Through this multifaceted regulation, *At*NPR1 preserves phloem functionality, restricts early pathogen-driven immune collapse, and maintains a balanced and resilient defense network (**Figure 9**). Together, these findings establish NPR1 as a central integrator of hormonal and structural immunity in citrus and highlight its potential in promoting durable HLB tolerance.

## Data Availability

The data presented in the study are deposited in the NCBI repository, accession number PRJNA1390723.
